# Acute Oral Toxicity Evaluations of Some Zinc(II) Complexes Derived from 1-(2-Salicylaldiminoethyl)piperazine Schiff Bases in Rats

**DOI:** 10.3390/ijms13021393

**Published:** 2012-01-27

**Authors:** Muhammad Saleh Salga, Hapipah Mohd Ali, Mahmood Ameen Abdulla, Siddig Ibrahim Abdelwahab

**Affiliations:** 1 Department of Chemistry, University of Malaya, Kuala Lumpur 50603, Malaysia; E-Mail: hapipah@um.edu.my; 2 Department of Molecular Medicine, University of Malaya, Kuala Lumpur 50603, Malaysia; E-Mail: mahmood955@yahoo.com; 3 Department of Pharmacy, University of Malaya, Kuala Lumpur 50603, Malaysia; E-Mail: siddigroa@um.edu.my

**Keywords:** synthesis, characterizations, acute toxicity, zinc complexes

## Abstract

The current study described the synthesis and the *in vivo* acute oral toxicity evaluations in *Sprague Dawley* rats. The compounds were characterized by elemental analyses, LC-MS, FTIR, ^1^H NMR, ^13^C NMR and UV-visible spectroscopy. In the acute toxicity study, a single administration of the compounds was performed orally to the rats at the single doses of 2000 mg/kg and they were then monitored for possible side effects, mortality or behavioral changes up to 14 days. The serum level of aspartate (AST), alanine aminotransferases (ALT), alkaline phosphate (ALP), triglyceride, high density lipoprotein (HDL), immunoglobulins (GAM) and the C-reactive proteins did not significantly change. The hematological indices white blood cells (WBC), haematocrit (HCT), red blood cells (RBC), mean corpuscular volume (MCV), mean corpuscular haemoglobin concentration (MCHC), and mean corpuscular hemoglobin (MCH) were within the normal range. The renal function indices examined were also within the reference range. Generally, the compounds exhibited low toxic effects as required for further *in vivo* therapeutic studies.

## 1. Introduction

Zinc has been shown to play an important role in wound healing, proper functioning of mucosal cells, reduction of reactive oxygen species (ROS) [1] and as a cofactor for metallo-enzymes [2]. Zinc deficiency or excess can lead to many metabolic disorders such as growth retardation, decreased spermatogenesis, dysgeusia, anosmia and anemia to meat, eggs, liver and oysters. Several studies were performed to determine the mechanisms for zinc balance and the effects of zinc excess on iron metabolism [3] with much emphasis on small molecular weight metal binding proteins [4]. Despite the biological importance of zinc, the safety of its compounds in many dietary supplements has remained an issue of debate. However, the interaction of zinc ions with certain Schiff base ligands has been studied due to their relevance in bio inorganic chemistry. For example, they form carbon-nitrogen bonds [5], which make them important intermediates in a number of enzymatic reactions [6–8]. Polydentate ligands, on the other hand, have been reported to exhibit potential activities in removing the undesirable effect of metal ion by deactivating either the carcinogenic metal or the enzyme required in order to protect the cells. The activities of various ligands were reported to have increased upon coordination with the metal ions; therefore, studies on novel metal-based compounds with therapeutic potential became an area of intense investigation in biomedical and inorganic chemistry [9–12]. However, metal ions are generally toxic at a high-dose level; therefore, to study the therapeutic potential of novel metal-based compounds; the acute toxicity level must first be evaluated. Moreover, the compounds containing piperazine moiety were reported to have shown various biological activities in many studies [13,14] and, specifically, the Schiff bases derived from piperazine compounds have been described to demonstrate various biological activities; for example, anthelmintic [15], antimicrobial [16,17], acetylcholinesterase inhibition [18], melanocortin-4-receptor (MC4-R) [19,20], drug designer [21] anti-PAF [22,23], anti-HIV [24,25] and anti-obesity [26] activities. However, the literature reveals no report on their toxicity class. This, therefore, prompted the present study to synthesize, characterize and evaluate for the first time the acute oral toxicity of some novel zinc(II) complexes derived from some 1-(2-salicylaldiminoethyl) piperazine Schiff bases.

## 2. Result and Discussion

### 2.1. Chemistry

**Scheme 1:**
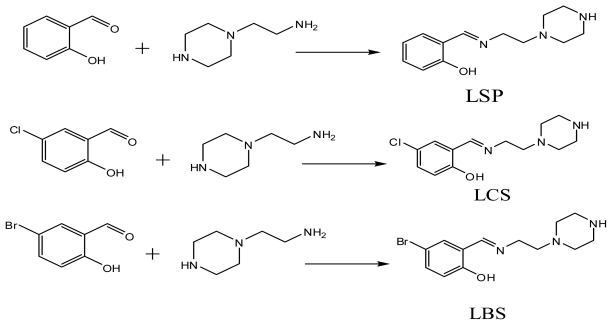
Reaction pathway for the Schiff bases.

**Scheme 2:**
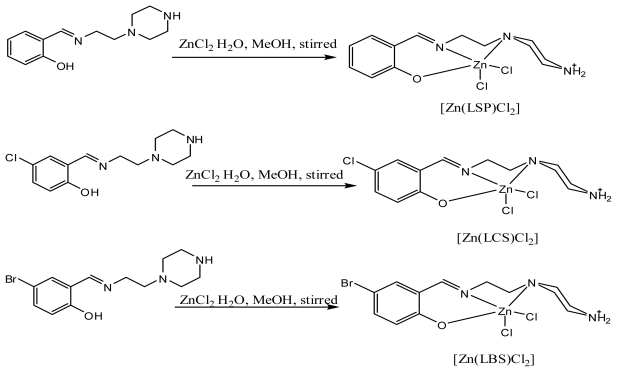
Reaction pathway for zinc complexes.

The reaction of 2-(piperazin-1-yl)ethanamine with some selected aldehydes resulted in the formation of the corresponding 1-(2-salicylaldiminoethyl)piperazines Schiff bases. The prepared Schiff bases (Scheme 1) were used to synthesize the novel complexes of zinc (II) chloride (Scheme 2). The compounds exhibited MS, NMR, IR and UV-Visible spectra consistent with the proposed structures which allowed the synthesized compounds to be recognized as 2-((2-(piperazin-1-yl)ethylimino) methyl)phenol-dichlorido-Zn-(II). [Zn(LSP)Cl_2_], 4-chloro-2-((2-(piperazin-1-yl)ethylimino)methyl) phenol-dichlorido-Zn-(II). [Zn(LCS)Cl_2_], 4-bromo-2-((2-(piperazin-1-yl)ethylimino)methyl)phenol-dichlorido-Zn-(II). [Zn(LBS)Cl_2_], respectively.

The IR spectra of the complexes displayed band regions at the wavelengths of 1,628, 1,624, and 1631 cm^−1^ for [Zn(LSP)Cl_2_], [Zn(LCS)Cl_2_] and [Zn(LBS)Cl_2_], respectively, which could be due to the characteristic iminic frequency [27,28]. These bands appeared at 1636 cm^−1^, 1631 cm^−1^ and 1616 cm^−1^ in the spectra of the free Schiff bases of the above-mentioned complexes correspondingly. In addition, the coordination of imine nitrogen to the zinc was further ascertained by the appearance of signal at the band regions 486 cm^−1^, 581 cm^−1^ and 578 cm^−1^ in the spectra of the corresponding complexes due to Zn-N bond [29] which is supported by the zinc-phenolate (Zn-O) [30] signals at 569 cm^−1^, 645 cm^−1^ and 631 cm^−1^ respectively. The proton NMR is also consistent with the IR spectral data, where the imine-zinc coordination was observed at 7.92 ppm, 8.02 ppm and 8.05 ppm in the spectra of [Zn(LSP)Cl_2_], [Zn(LCS)Cl_2_] and [Zn(LBS)Cl_2_], respectively. These signals initiated from 7.28 ppm, 7.53 ppm and 7.68 ppm in the spectra of the free Schiff bases of the corresponding complexes. This supposition was supported by the ^13^C NMR spectra which showed imine carbon at 162.6 ppm, 164.2 ppm and 165.2 ppm respectively due to complexation. The phenolate carbon atoms also appeared at 161.5 ppm, 159.5 ppm and 158.4 ppm in the respective order of the complexes mentioned above [31]. To further elucidate the structure of the complexes, UV-visible spectra were recorded using DMSO. The spectra of the complexes exhibited two absorption band maxima each at 279 nm, 204 nm and 267 nm for [Zn(LSP)Cl_2_], [Zn(LCS)Cl_2_] and [Zn(LBS)Cl_2_] respectively. This could be afforded to the π-π* electronic transitions [32,33] the phenolic ring. The other absorptions noticeable to 351 nm, 362 nm and 382 nm can be due to ligand to metal charge transfer [34,35].

### 2.2. Acute Toxicity Study

The analysis of the toxicity level of chemical compounds is the most important step required for further biological studies [36]. The toxicity level of the zinc complexes derived from 1-(2-salicylaldiminoethyl)piperazines were evaluated at the maximum dose of 2000 mg/kg/body weight. The compounds were administered orally to the 24 h fasted rats and monitored closely after every 30 min up to 8 h of post treatment. It was observed that the compounds did not cause any gross behavioral alterations like convulsion, dizziness or respiratory distress. No mortality was recorded for the period of 14 days, which indicate that the lethal dose of the compounds is above 2000 mg/kg body weight in rats and that the compounds can be considered to be less harm at this dose.

### 2.3. Body and Organ Weight Changes

**Figure 1:**
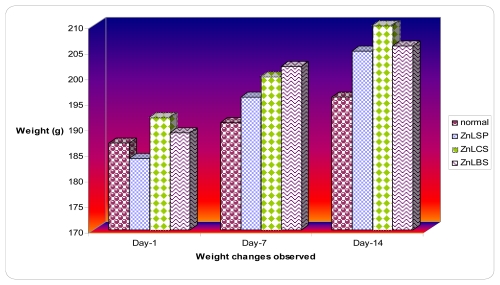
Effects of zinc complexes on the body weights.

**Figure 2:**
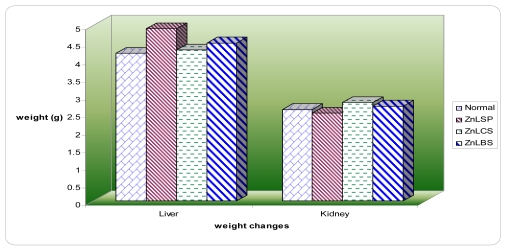
Effects of zinc complexes on the organ weights.

The animals treated with the zinc complexes for two weeks had manifested an increase in body weight slightly above the animals in the control group (Figure 1). The target organs such as liver and kidney of both the control and the treatment group did not exhibit any change in color or texture, and the weight of these organs was not significantly (*P* < 0.05) affected by the zinc complexes (Figure 2). This also demonstrated the less toxic effect [37] of the compounds.

### 2.4. Effects of Zinc Complexes on the Biochemical Indices

The oral administration of the zinc complexes for two weeks did not cause any significant changes in the biochemical parameters such as renal function indices like creatinine, urea, anion gap sodium and carbon dioxide. Both the normal rats and the rats treated with zinc complexes had manifested the level of biochemical indices within the normal range (Table 1). However, the liver enzymes AST, ALT, ALP and triglyceride rose significantly in the rats that received ZnLSP, compared to the rats administered with the complexes ZnLCS and ZnLBS and the normal rats. This can be attributed to the damage in the liver cells [38] due to low toxic effect of the compounds. This is reduced in the analogue complexes that contained ring substituents in their structures, thus displaying the influence of ring substituents on the activity of the compounds [39,40]. Other liver indices like total protein count, albumin, globulins, total cholesterol, total and conjugated bilirubin did not significantly change at the dose of 2000 mg/kg/body weight (Table 2). A similar result was obtained in the acute toxicity evaluations of the free ligands in our previous study [27].

The hematological profile of the rats treated with zinc complexes did not significantly differ in the red blood cell (RBC), mean corpuscular volume (MCV) and mean corpuscular hemoglobin (MCH). Furthermore, the values of the biomarkers like hematocrit (HCT), RDW and platelet in the treated rats are comparable with those in the normal rats, except the biomarker MCHC which showed inconsistent results in the treatment groups. However, for ZnLCS and ZnLBS, the values obtained are within the physiological ranges [41] for rats (Table 3). The value obtained for MCHC in the rats treated with the complex ZnLSP is below that of the normal rats and there is no interpretation from the literature for this.

The acute phase immunoglobulins’ G, A and M, the complements 3 and 4, and the level of C-reactive protein did not significantly differ between the normal, and the treated rats in both gender. This indicates that the complexes did not interfere with the immune system of the treated rats [42] (Table 4).

## 3. Experimental

### 3.1 Chemistry

2-(piperazin-1-yl)ethanamine, salicylaldehyde, 5-chlorosalicylaldehyde, and 5-bromosalicylaldehyde were used without further purification. Methanol, absolute ethanol, dimethylsulfoxide (DMSO) and all other solvents were of analytical grade. Spectroscopic grade DMSO-d_6_ was used for ^1^H and ^13^C NMR. All the chemicals used were purchased from Sigma Aldrich (Kuala Lumpur, Malaysia) and used without further purification. Mass spectra were determined using ABI 4800 Maldi TOF/TOF mass spectrophotometer (BIDMC Genomics, Proteomics and Bioinformatics Core, Boston, MA, USA) (LC-MS, ESI, 125.0 V); IR spectra was recorded at the wavelength range from 4000–400 cm^−1^ using a Perkin Elmer 783 spectrophotometer; NMR spectra was obtained on a ECA400 FT-NMR spectrophotometer using TMS as internal standard, UV-visible spectra was recorded on an UV-1650PC model UV-visible spectrophotometer.

### 3.2. Schiff Bases

The Schiff bases (LSP, LCS and LBS) were prepared according to the reported general procedure [43] described below with some modifications.

To the ethanolic solution (25 mL) of (2-piperazin-1-yl)ethanamine (2.58 g, 20 mmol), salicylaldehyde (2.44 g, 20 mmol) taken in ethanol (25 mL) was added with stirring. The resulting solution was refluxed for three hours, cooled and concentrated to give a red gel. The gel became hygroscopic solid after seven days under vacuum. The solid product is then dissolved in methanol by heating to 55 °C. While hot, few drops of diethyl ether were added and yellow solid appeared which was collected by filtration. Recrystallization was performed in ethanol-water mixture. The same procedure was followed in the preparation of LCS and LBS Schiff bases.

### 3.3. Complexes

#### 3.3.1. 2-((2-(piperazin-1-yl)ethylimino)methyl)phenol-dichlorido-Zn-(II): [Zn(LSP)Cl_2_]

Stoichiometric amount of Zinc (II) chloride (0.14 g, 1 mmol) in methanol (25 mL), was added to an equimolar quantity of the appropriate Schiff base (1 mmol) dissolved in the same solvent (25 mL) at room temperature and followed with few drops of potassium hydroxide. A yellow precipitate was produced upon stirring. The precipitate filtered, washed with distilled water and dried in the vacuum for further analysis. The same method was applied in the synthesis of [Zn(LCS)Cl_2_] and [Zn(LBS)Cl_2_]. C_13_H_18_Cl_2_N_3_ZnO: yield; (0.15 g 40.6%). Anal. Cal. C, 66.9; H, 8.21; N, 18.01. Found: C, 65.82; H, 7.76; N, 17.97%. *m/z*: 369.03, 367.04, 371.01. IR (KBr disc, 4000–400 cm^−1^) selected bands: *ν* (N–H), 3442; *ν* (C–H) alip., 2825; *ν* (C=N), 1628; *ν* (C–C) arom., 1468; *ν* (C–N), 1152; *ν* (C–H) arom.768; *ν* (M–O), 569; *ν* (M–N), 486. ^1^H NMR (400 MHz, DMSO-*d*6) *δ* ppm: 7.92 (s, 1H, –C=N–); 2.66–3.45 (t, 2H, C_aliph_); ^13^C NMR (100 MHz, DMSO-*d*6) *δ* ppm: 46.5 (CH_2_); 34.51 (CH_2_); 36.5 (CH_2_); 39.4 (CH_2_); 122.8 (armC); 161.5 (CO); 162.6 (C=N); 117.6 (armC); 125.7 (armC). UV-vis (DMSO), *λ*_max_ (ɛ, mol^−1^·L cm^−1^): 279 nm (2647.49, π-π*), 351 nm (2811.20, LMCT).

#### 3.3.2. 4-chloro-2-((2-(piperazin-1-yl)ethylimino)methyl)phenol-dichlorido-Zn(II): [Zn(LCS)Cl_2_]

C_13_H_17_N_3_Cl_3_O_2_Zn: yield; (0.19 g, 47%). Anal. Cal. C, 58.31; H, 6.78; N, 15.69. Found: C, 57.97; H, 5.94; N, 15.27. m/z: 402.98, 400.98, 404.97. IR (KBr disc, 4000–400 cm^−1^) selected bands: *ν* (N–H), 3459; *ν* (C–H) alp., 2966; *ν* (C=N), 1624; *ν* (C-C) arom., 1466; *ν* (C–N), 1172; *ν* (C–H) arom.704; *ν* (M–O), 645; *ν* (M–N), 581. ^1^H NMR (400 MHz, DMSO-*d*6) *δ* ppm: 8.02 (s, 1H, –C=N–); 2.56–3.48 (t, 2H, C_aliph_); ^13^C NMR (100 MHz, DMSO-*d*6) *δ* ppm: 45.6 (s, 1 CH_2_); 34.8 (s, 1 CH_2_); 34.9 (s, 1 CH_2_); 123.2 (s, 1 armCH_2_); 159.5 (s, 1 CO); 164.2 (s, 1 C=N); 116.2 (s, 1 armCH_2_); 123.6 (s, 1 CH_2_). UV-vis (DMSO), *λ*_max_ (ɛ, mol^−1^ L·cm^−1^): 204 nm (937.9, π-π*), 326 nm (3989.7, LMCT)

#### 3.3.3. 4-bromo-2-((2-(piperazin-1-yl)ethylimino)methyl)phenol-dichlorido-Zn-(II): [Zn(LBS)Cl_2_]

C_13_H_17_N_3_BrCl_2_O_2_Zn: yield; (0.32g 71.3%). Anal. Cal. C, 34.89; H, 3.83; N, 9.39. Found: C, 34.82; H, 3.66; N, 8. 98. m/z: 446.93, 448.93, 450.92. IR (KBr disc, 4000–400 cm^−1^) selected bands: *ν* (N–H), 3448; *ν* (C–H) alp., 2966; *ν* (C=N), 1631; *ν* (C–C) arom., 1466; *ν* (C–N), 1169; *ν* (C–H) arom. ) 686; *ν* (M–O), 631; *ν* (M–N), 578. ^1^H NMR (400 MHz, DMSO-*d*6) *δ* ppm: 8.05 (s, 1H, –C=N–); 2.52–3.43 (t, 2H, C_aliph_); ^13^C NMR (100 MHz, DMSO-*d*6) *δ* ppm: 46.6 (s, 1 CH_2_); 34.7 (s, 1 CH_2_); 35.1 (s, 1 CH_2_); 122.1 (s, 1 CH_2_); 158.4 (s, 1 CO); 165.2 (s, 1 C=N); 114.4 (s, 1 CH_2_); 124.5 (s, 1 CH_2_). UV-vis (DMSO), *λ*_max_ (ɛ, mol^−1^·L cm^−1^): 267 nm (1939.17, π-π*), 382 nm (1564.26, LMCT)

### 3.4. Animals

Adult *Sprague Dawley* rats of 8–9 weeks old weighed 180–200 g were obtained from Animal House, Faculty of Medicine, University of Malaya (Kuala Lumpur, Malaysia). The animals were housed in animal room at temperatures 22 ± 3 °C and 12 h dark period. After one-week acclimatization, rats were distributed into four groups of ten rats each (five males and five females, labeled as control and treated) and maintained on standard pellet food and purified drinking water. All animals received human care according to the criteria outlined in the “Guide for the Care and Use of Laboratory Animals” prepared by the National Academy of Sciences and published by the National Institute of Health.

### 3.5. Acute Toxicity Test

Acute toxicity evaluations were carried out on rats according to the reported method with some modifications [44]. *Sprague Dawley* rats of both genders were divided into experimental and control groups (10 rats per group of five males and five females each). The study was executed at a single oral dose of 2000 mg/kg body weight, in 5 mL/kg volume. The control group was treated with distilled water. The experimental group was fasted for 24 h before the administration of the compound but allowed access to distilled water. The animals were further denied access to food for 2 h of post treatment in order to examine the possible adverse effects of the compounds such as behavioral adjustments, autonomous released of mucus, dizziness restlessness or mortality.

### 3.6. Statistical Analysis

The results were analyzed using one-way analysis of variance (ANOVA) and expressed as mean ± SEM. Probability values of *P* < 0.05 was considered statistically significant.

## 4. Conclusion

In conclusion, the results of this study showed that the zinc complexes derived from the Schiff bases 2-(2-(piperazin-1-yl)ethylimino)methyl)phenol, 4-chloro-2-(2-(piperazin-1-yl)ethylimino)methyl) phenol and 4-bromo- 2-(2-(piperazin-1-yl)ethylimino)methyl) phenol have fewer toxic effects based on the insignificant changes observed in the behavioral, hematological, immunological and biochemical parameters. However, a decrease in the activity of liver and some hematological indices was noted, which require further study to fully ascertain the safety of the compounds at high doses.

## Figures and Tables

**Table 1:** Effect of zinc complexes on the renal functions.

Indices	Normal	[Zn(LSP)Cl_2_]	[Zn(LCS)Cl_2_]	[Zn(LBS)Cl_2_]
Sodium	139.5 ± 2.6	139.2 ± 2.4	138.2 ± 2.7	138.5 ± 2.9
Potassium	5.05 ± 0.8	5.50 ± 0.6	4.70 ± 0.9	5.100 ± 0.7
Chloride	103.8 ± 1.3	104.6 ± 2.3	105.1 ± 1.2	102.3 ± 1.7
CO_2_	23.9 ± 2.1	22.2 ± 2.6	21.3 ± 3.2	23.10 ± 3.3
Anion gap	17.4 ± 1.2	18.5 ± 0.7	16.5 ± 3.1	18.50 ± 3.5
Urea	6.10 ± 1.3	7.40 ± 0.6	7.60 ± 1.7	9.300 ± 2.6
Creatinine	42.5 ± 1.9	28.4 ± 17	40.8 ± 1.9	50.50 ± 1.9

**Table 2:** Effects of zinc complexes on the liver functions.

Indices	Normal	[Zn(LSP)Cl_2_]	[Zn(LCS)Cl_2_]	[Zn(LBS)Cl_2_]
Total protein	70.5 ± 3.6	87.3 ± 4.9	75.5 ± 3.2	81.8 ± 2.6
Albumin	59.5 ± 2.4	68.6 ± 4.1	62.5 ± 3.2	69.3 ± 0.5
Globulin	59.5 ± 3.4	69.5 ± 4.9	61.9 ± 2.2	65.3 ± 2.4
Alk. Phosphate	59.30 ± 11.3	81.30 ± 10.8	82.3 ± 11.3	92.80+12.5
ALT	49.8 ± 7.2	61.0 ± 3.6	57.8 ± 3.4	55.3 ± 4.9
AST	259.8 ± 12.7	292.3 ± 10.6	278.5 ± 9.8	281.5 ± 12.6
Total bilirubin	6.25 ± 0.5	7.50 ± 0.7	6.88 ± 0.8	7.32 ± 0.8
C.bilurubin	3.61 ± 0.9	5.83 ± 1.3	3.85 ± 1.4	4.22 ± 2.1
Triglyceride	0.45 ± 0.1	0.60 ± 0.4	0.30 ± 0.05	0.80 ± 0.8
Total cholesterol	2.20 ± 0.3	3.70 ± 0.5	3.30 ± 0.1	3.6 0± 0.4
HDL	1.53 ± 0.4	1.47 ± 0.2	1.39 ± 0.4	1.50 ± 0.6

**Table 3:** Effects of zinc complexes on the hematological indices.

Indices	Normal	[Zn(LSP)Cl_2_]	[Zn(LCS)Cl_2_]	[Zn(LBS)Cl_2_]
HGB	151.3 ± 11.0	162.3 ± 11.8	158.3 ± 12.2	163.3 ± 13.5
HCT	0.540 ± 0.21	0.980 ± 0.03	0.830 ± 0.01	0.920 ± 0.04
RBC	7.90 ± 0.4	8.10 ± 0.3	8.60 ± 0.3	8.80 ± 0.7
MCV	65.2 ± 2.4	69.3 ± 1.5	67.9 ± 1.8	76.9 ± 1.8
MCH	17.9 ± 0.9	18.1 ± 0.3	18.9 ± 0.6	18.6 ± 0.5
MCHC	291.2 ± 2.3	260.5 ± 4.4	302.3 ± 5.6	269.3 ± 4.5
RDW	15.8 ± 1.4	17.5 ± 1.2	16.6 ± 1.5	17.9 ± 2.1
WBC	10.7 ± 3.1	11.3 ± 0.4	12.9 ± 4.4	12.4 ± 1.8
Platelet	654.3 ± 9.5	846.6 ± 8.5	689.3 ± 9.7	708.3 ± 9.4

**Table 4:** Effects of zinc complexes on the immunological indices.

Indices	Normal	[Zn(LSP)Cl_2_]	[Zn(LCS)Cl_2_]	[Zn(LBS)Cl_2_]
ImmunoglobulinG	933.4 ± 2.3	933.8 ± 4.2	933.5 ± 3.6	933.9 ± 4.8
Immunoglobulin A	97.5 ± 2.2	89.6 ± 3.7	98.7 ± 4.4	99.2 ± 4.3
Immunoglobulin M	43.9 ± 7.9	42.3 ± 3.5	52.7 ± 8.3	63.5 ± 9.6
Complement 3	96.8 ± 2.3	96.2 ± 1.2	96.6 ±3.2	96.9 ±1.7
Complement 4	29.8 ±2.1	52.9 ±3.2	57.2 ± 4.2	57.9 ± 4.2
C-reactive Protein	0.42 ± 0.3	0.34 ± 0.5	0.45 ± 0.2	0.53 ± 0.2
